# Intrauterine growth restriction alters kidney metabolism at the end of nephrogenesis

**DOI:** 10.1186/s12986-023-00769-6

**Published:** 2023-11-21

**Authors:** Sheng-Yuan Ho, Merryl Esther Yuliana, Hsiu-Chu Chou, Chung-Ming Chen

**Affiliations:** 1 Graduate Institute of Clinical Medicine, College of Medicine, Taipei Medical University, Taipei, Taiwan; 2 Department of Pediatrics, Tri-Service General Hospital, National Defense Medical Center, Taipei, Taiwan; 3 International Ph.D. Program in Medicine, College of Medicine, Taipei Medical University, Taipei, Taiwan; 4 Faculty of Medicine, Christian University of Indonesia, Jakarta, Indonesia; 5 Department of Anatomy and Cell Biology, School of Medicine, College of Medicine, Taipei Medical University, Taipei, Taiwan; 6 Department of Pediatrics, School of Medicine, College of Medicine, Taipei Medical University, Taipei, Taiwan; 7 Department of Pediatrics, Taipei Medical University Hospital, Taipei, Taiwan

**Keywords:** Uteroplacental insufficiency, Intrauterine growth restriction, Kidney, Metabolomics, Nephrogenesis

## Abstract

**Background:**

This study investigated the effect of uteroplacental insufficiency (UPI) on renal development by detecting metabolic alterations in the kidneys of rats with intrauterine growth restriction (IUGR).

**Methods:**

On gestational day 17, pregnant Sprague Dawley rats were selected and allocated randomly to either the IUGR group or the control group. The IUGR group received a protocol involving the closure of bilateral uterine vessels, while the control group underwent a sham surgery. The rat pups were delivered on gestational day 22 by natural means. Pups were randomly recruited from both the control and IUGR groups on the seventh day after birth. The kidneys were surgically removed to conduct Western blot and metabolomic analyses.

**Results:**

IUGR was produced by UPI, as evidenced by the significantly lower body weights of the pups with IUGR compared to the control pups on postnatal day 7. UPI significantly increased the levels of cleaved caspase-3 (*p* < 0.05) and BAX/Bcl-2 (*p* < 0.01) in the pups with IUGR. Ten metabolites exhibited statistically significant differences between the groups (*q* < 0.05). Metabolic pathway enrichment analysis demonstrated statistically significant variations between the groups in the metabolism related to fructose and mannose, amino and nucleotide sugars, and inositol phosphate.

**Conclusions:**

UPI alters kidney metabolism in growth-restricted newborn rats and induces renal apoptosis. The results of our study have the potential to provide new insights into biomarkers and metabolic pathways that are involved in the kidney changes generated by IUGR.

**Supplementary Information:**

The online version contains supplementary material available at 10.1186/s12986-023-00769-6.

## Introduction

Intrauterine growth restriction (IUGR) affects 10–23.8% of pregnancies worldwide and is a major predictor of mortality and poor prognosis for fetuses and neonates [1]. IUGR increases the risk of developmental programming of chronic kidney disease, hypertension, and renal tubular damage in children and adults [2, 3]. Uteroplacental insufficiency (UPI) is a leading cause of these diseases [4, 5]. To investigate intrauterine growth restriction in developed countries, bilateral uterine vessel ligation is a superior model to the protein-restricted model, which relies on changes in placental blood flow in animal experiments [6]. Previous studies have shown that the possible pathogenesis of IUGR-induced chronic kidney disease or nephron loss is related to renal oxidative stress, autophagy, and apoptosis in rats with IUGR induced by protein restriction [7]. In one study, term infants with IUGR exhibited subclinical renal damage, particularly in the proximal tubules, as indicated by higher levels of urinary neutrophil gelatinase–associated lipocalin and microalbumin [8]. The proximal tubules in the renal cortex have the highest mitochondrial content in the kidney. Therefore, the proximal tubules are highly vulnerable to hypoxia and rely on mitochondrial oxidative metabolism to meet energy requirements. Integrity of mitochondria is maintained through the integration of metabolism and apoptosis, which involves the proapoptotic Bcl-2 family and the caspases family [9]. However, previous studies on IUGR have yielded inconclusive results regarding whether apoptosis increases during kidney development [10, 11]. Human kidney development is completed in utero within 36 weeks [12], whereas rat kidney development continues for an additional 6–8 days after birth [13, 14]. Therefore, the programming of renal development can be studied using the neonatal rat model. Considering the available evidence, we focused on the renal cortical cell apoptotic and metabolic pathways in a UPI-induced IUGR rat model.

Metabolomics has become a useful discipline within perinatal medicine in recent years. Metabolomics is the study of changes in metabolite composition, which are caused by specific pathophysiological states, through high-throughput analysis of the metabolites in metabolic pathways [15]. The translational capacity of metabolomics may enable the identification of new biomarkers for IUGR [16]. Whether UPI-induced IUGR increases apoptosis as it does in animal models of protein restriction-induced IUGR remains unknown. According to our review of the literature, no studies have examined the differences in renal metabolism caused by IUGR. This study aimed to examine the impact of UPI on renal development by identifying metabolic alterations in the kidneys of rats exhibiting IUGR. Our hypothesis posited that the rats subjected to UPI-induced IUGR would have heightened cellular apoptosis and modified metabolic pathways in the renal cortex.

## Materials and methods

### Animal model and experimental groups

The research was conducted in adherence to the protocols outlined by the Animal Care Use Committee of Taipei Medical University (LAC-2022-0120). The study utilized Sprague Dawley rats that were time-dated for pregnancy. These rats weighed between 180 and 250 g and were 6–8 weeks old. The rats were at 14 days of gestation at the time of purchase, and they were obtained from BioLASCO in Taiwan. During the preexperimental period, the experimental rats were given food and water ad libitum and housed in a room with a temperature range of 20–25 °C and a relative humidity range of 40–60% for a duration of one week. A light–dark cycle of 12:12 h was maintained. On day 17 of gestation, general anesthesia was induced in the animals with isoflurane, and the experimental and control groups underwent bilateral uterine vessel ligation and sham surgery, respectively. It was necessary to execute a midline laparotomy to expose the uterine horns and their blood vessels. The ligation locations were chosen based on the scientific literature [6]. To maintain blood flow from both the iliac arteries and ovaries, the uterine vessels were ligated in the midsection of each uterine horn to increase the likelihood of fetal survival and decrease the probability of a partial miscarriage. The sham surgery performed on the control group did not involve ligation. After repositioning the uterus within the abdominal cavity, lidocaine was administered at the incision site [6, 17]. On day 22 of gestation, every rat offspring was born naturally. Litter size was adjusted based on recommendations for testing maternal effects on reproductive/developmental toxicity [18, 19]. Within 12 h of birth, the litters in the control and UPI groups were aggregated within groups and reassigned at random to the mothers who delivered them in the same group. After the pups were euthanized on postnatal days 0 and 7, the litter size of the control and IUGR groups was reduced to nine and five pups, respectively, to ensure equal access to breast milk. The sham-operated dams had three litters of 39 pups. The UPI-induced group consisted of four dams and 26 pups in four litters. 12 pups from sham-operated dams and 8 pups from UPI-induced dams were obtained on postnatal day 0. The remaining pups were fed by three sham-operated dams and two UPI-induced dams, respectively. On postnatal day 7, 12 pups from sham-operated dams and 8 pups from UPI-operated dams were examined. On the seventh postnatal day, rats of both sexes were chosen at random from each group. A single kidney was removed and dissected by cutting it longitudinally. The kidney was then placed under a magnifying glass, the cortex of the kidney was dissected from the medulla with a sharp scalpel, and kidney tissue was obtained for Western blot and metabolomic analysis.

### Western blot analysis

Samples of kidney tissue were trypsinized and flushed with phosphate-buffered saline (PBS) prior to resuspension (1500 rpm, 7 min). After aspiration of PBS, 100 L of lysate buffer and protease inhibitor were added. After 30 min on ice, the sample was centrifuged for 20 min at 12,000 rpm and 4 °C in a microcentrifuge. The centrifuge tubes were placed on ice. The pellet was discarded while the supernatant was aspirated and deposited in a separate tube on ice. Thirty grams of protein was resolved by 12% sodium dodecyl sulfate–polyacrylamide gel electrophoresis, electroblotted, and transferred to polyvinylidene difluoride membranes (ImmobilonP, Millipore, Bedford, MA, USA). Following blocking with 5% nonfat dried milk, the membranes were incubated with cleaved caspase 3 (1:1000, #9664, Cell Signaling Technology), Bax (1:750, B-9 sc-7480, Santa Cruz Biotechnology, Dallas, TX, USA), Bcl-2 (1:750, C-2 sc-7382, Santa Cruz Biotechnology), and anti-β-actin (1:1,000, C4 sc-47778, Santa Cruz Biotechnology). Subsequently, the samples were subjected to incubation with horseradish peroxidase–conjugated goat anti-mouse antibodies (Pierce Biotechnology, Rockford, IL, USA). The detection of protein bands was performed utilizing the BioSpectrum AC Imaging System (UVP, Upland, CA, USA).

### Metabolomics

#### Sample preparation

The kidney tissue samples were obtained by utilizing 100 μL of methanol solution (Macron Chemicals, Center Valley, PA, USA) and H_2_O (Cat# W4502, Sigma‒Aldrich, St. Louis, MO, USA; at a ratio of 7:3 v:v). Following two freeze‒thaw cycles, the samples were subjected to vortexing. Following the centrifugation of each sample at 4 °C and 12,000 × g for a duration of 15 min, the supernatant was collected, rapidly dried under vacuum, and dissolved in 0.3 mL of a 50:50 mixture of H2O and CH3CN.

#### Liquid chromatography/mass spectrometry analysis

The chromatographic separation was conducted utilizing a Waters ACQUITY ultraperformance liquid chromatography (UPLC) system. A UPLC BEH C18 guard column (1.7 μm, 5 mm) was employed as the reversed-phase column. The analytical column (1.7 μm, 2.1 × 100 mm) was kept at a temperature of 45 °C. The mobile phase employed for linear gradient separation comprised two components: (A) water with 0.1% formic acid and (B) acetonitrile supplemented with 0.1% formic acid.

A SYNAPT G2 quadrupole time-of-flight mass spectrometer (Waters MS Technologies, Manchester, UK) was utilized to conduct mass spectrometry (MS) analysis. The mass spectrometer was configured as follows: negative ion mode, capillary voltage of 2 kV, source temperature of 120 °C, desolvation gas N2 at 900 L/h at 550 °C, cone gas N2 at 15 L/h, capillary voltage of 2.8 kV, cone voltage of 40 V, and time-of-flight mass spectrometry (TOF MS) scan range of 50–1000 m/z. The Waters MS acquisition mode was utilized, and the data acquisition rate and interscan latency were 1.2 and 0.02 s, respectively. Simultaneous recording of the exact masses of all molecules was accomplished by rapidly cycling between two functions. The first function gathered data with a low impact energy of 4 eV for the collision cell trap and 2 eV for the collision cell transfer, while the second function gathered data with a modulated transfer collision energy of 15–25 eV. All analyses were conducted with a lockspray to guarantee precision and reproducibility. As the lockmass, we utilized leucine-enkephalin (*m/z* = 556.2771) at a concentration of 1 ng/L and a flow rate of 5 L/min. The data were collected in continuous mode with a 20-s lockspray interval. For all data collection, Waters MassLynx MS software (version 4.1) was employed.

### Analysis of metabolomic data

Progenesis QI software (Waters, Milford, MA, USA) was used to analyze the raw mass data generated using a Waters SYNAPT G2 for peak detection, extraction, alignment, and integration; the parameters were adjusted for each processing step. We regarded a difference of at least 1.2-fold between the median intensities of the two sample groups to indicate differential metabolite levels. The compounds associated with the pathway were compared to those listed in the Human Metabolome Database. Compound identification was performed using Progenesis QI, resulting in an overall score of 40 based on mass accuracy and isotope patterns for compound prediction. A minimum score of 36 or higher was utilized. The compounds were subjected to pathway enrichment analysis using MetaboAnalyst 5.0 and were then compared with the Kyoto Encyclopedia of Genes and Genomes (KEGG).

The quality control pool referencing method was applied to all intact MS samples, and it was seen that they aligned with the reference at a minimum of 90%, indicating the dependability of the 1.7-m ACQUITY Premier CSH Phenyl-Hexyl Column. The retention time and *m/z* pairs of distinct ions were combined through the use of adducts and isotope deconvolution techniques. This process allowed for the aggregation of the abundance of unique ions and the creation of distinctive characteristics, characterized by their retention time and *m/z* pairs, which are representative of unidentified metabolites. The data underwent normalization using Progenesis QI for all the characteristics. The abundance ratio of feature ions in a specific run to their corresponding value in the normalized reference was calculated by measuring each feature ion in every run. The data underwent Log10 transformations using Progenesis QI software to achieve normal distributions for each procedure and sample. Scalar estimation was then applied to change the log10 distributions for the purpose of normalizing the reference data.

### Statistical analysis

The data are presented as the mean ± standard deviation. Statistical significance was determined when the *p* value was less than 0.05. One-way analysis of variance was conducted using Progenesis QI to determine if there were any statistically significant differences between the IUGR and control groups. The pooled abundance data were analyzed using Progenesis QI to generate the fold change (FC) criterion, with a FC ≥ 1.2 being considered significant [20–22]. Volcano plots were employed as a graphical representation to depict dysregulated metabolites, with the log2-fold change plotted against the negative logarithm of the *p* value. Tentative and putative annotations in Progenesis QI were established by considering accurate mass measurements with an error of less than 5 ppm, similarity in isotope distribution, and manual matching of fragmentation spectra (if applicable) with databases such as the Human Metabolome Database [23], Metlin [24], MassBank [25], and the National Institute of Standards and Technology database.

The MS data were subsequently employed for relative quantification through the utilization of the mixOmics package in R programming language. To reduce the dimensionality of the data, they were exported for unsupervised principal component analysis (PCA). To visualize data clustering and identify substantially different metabolites, we performed a supervised analysis, namely, partial least-squares discriminant analysis (PLS-DA), and obtained variable importance in projection (VIP) scores. Each metabolite was compared between the groups using a univariate Student’s *t* test in MetaboAnalyst 5.0, and the Benjamini–Hochberg method was performed to adjust the *p* values for multiple testing with consideration of a 5% false discovery rate (*q* value). The metabolites with a VIP > 1 and *q* < 0.05 were considered significantly differing metabolites. Threefold cross-validation was performed to evaluate the goodness of fit of the PLS-DA model on the basis of *R*^2^ and *Q*^2^ values. Pathway analysis in MetaboAnalyst was performed using Fisher’s exact test to calculate the probability of finding a certain number of metabolites of a biological term of interest in a given list of compounds based on the KEGG [26].

## Results

There were three dams in the sham-operated group, and three litters of 39 pups were delivered. There were four dams in the UPI-induced group, and a total of 26 pups were delivered in four litters. On postnatal day 0, a total of 12 pups (five males and seven females) from sham-operated dams and eight pups (four males and four females) from UPI-induced dams were collected for examination. The remaining pups were distributed among three dams in the sham-operated group and two dams in the UPI-induced group for feeding. On postnatal day 7, we obtained a total of 12 pups (six males and six females) from the dams that underwent sham surgery and eight pups (three males and five females) from the dams that underwent UPI-induced surgery. There was no statistically significant difference in the pup sex ratio between the sham-operated group and UPI-induced group on postnatal day 0 and postnatal day 7 (*p* = 0.714 on postnatal day 0 and *p* = 0.582 on postnatal day 7).

### UPI induced lower body and kidney weights in growth-restricted newborn rats on postnatal days 0 and 7

IUGR was confirmed by the considerably lower mean birth weight of rats with IUGR compared to control rats (Table 1). The pups with IUGR (n = 8) had substantially lower body weights on postnatal day 7 (14.50 ± 0.55 vs. 16.90 ± 1.39 g; *p* < 0.001) compared to the control pups (n = 12). On postnatal day 7, the kidney weights of pups with IUGR were markedly lower than those of pups without IUGR (0.189 ± 0.016 vs. 0.223 ± 0.025 g; *p* < 0.01). However, the ratio of kidney to body weight did not differ significantly between the two groups.

**Table 1:** The ratio of kidney weight to body weight on postnatal days 0 and 7 in the control and IUGR groups

Group	*n*	Body weight (g)	Kidney weight (g)	Kidney: body weight (%)
*Postnatal day 0*				
Control	12	6.40 ± 0.56	0.072 ± 0.012	1.13 ± 0.20
IUGR	8	5.38 ± 0.88^**^	0.048 ± 0.016^**^	0.91 ± 0.32†
*Postnatal day 7*				
Control	12	16.90 ± 1.39	0.223 ± 0.025	1.33 ± 0.19
IUGR	8	14.50 ± 0.55^***^	0.189 ± 0.016^**^	1.31 ± 0.13

On postnatal day 0, the sham-operated and UPI-induced dams had three and four litters, respectively. On postnatal day 7, the offspring number was reassigned to three and two in the sham-operated and UPI-induced groups, respectively. Values are means ± standard deviations. ^**^p < 0.01, ^***^p < 0.001 versus control group at each postnatal age. †*p* = 0.078 versus control group on postnatal day 0. IUGR: intrauterine growth restriction

### UPI induced higher cleaved caspase-3 and Bax/Bcl-2 levels in growth-restricted newborn rats

**Fig. 1:**
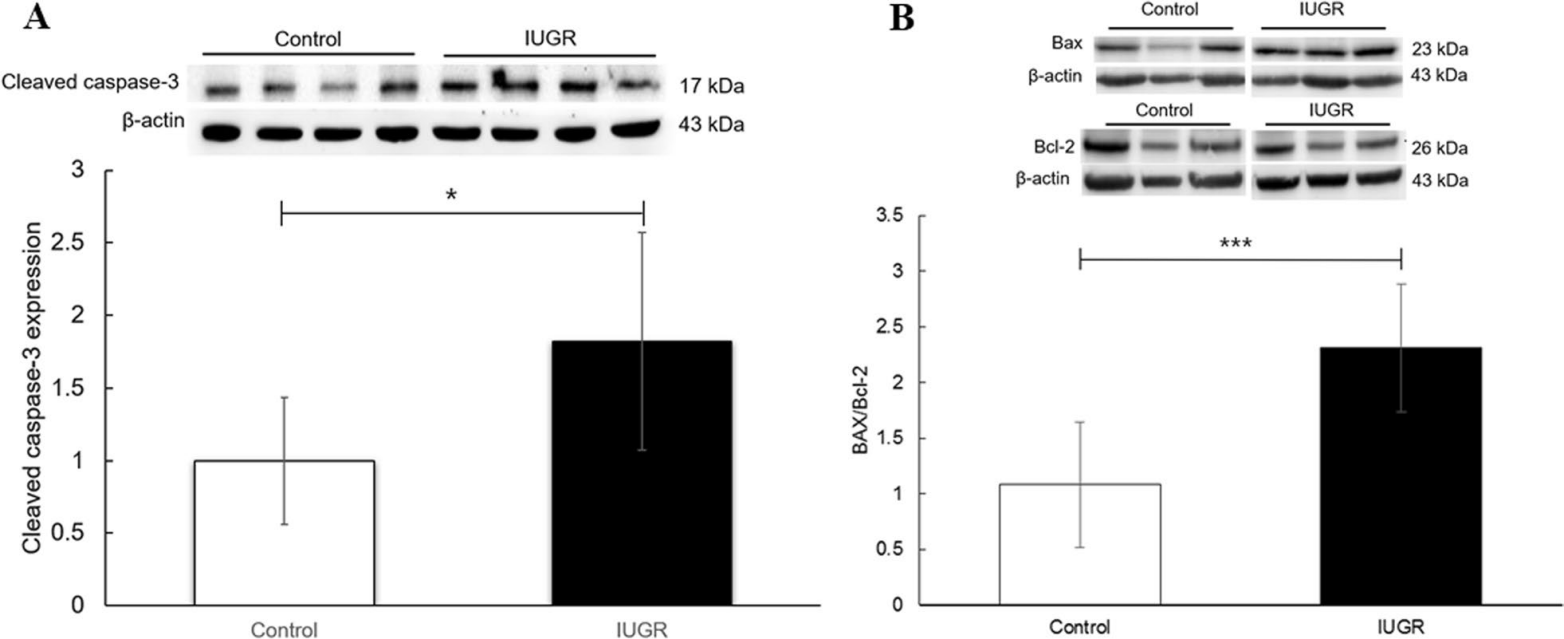
Representative Western blots of **A** cleaved caspase-3 and **B** Bax/Bcl-2 in the renal cortex of rats on postnatal day 7 from the control and intrauterine growth restriction (IUGR) groups. The mean levels of cleaved caspase-3 and Bax/Bcl-2 were substantially higher in experimental rats than in control rats. Data are shown as the mean ± standard deviation. *p < 0.05, ***p < 0.001 (n = 4)

Stewart et al. demonstrated increased apoptosis of renal cells in rats with IUGR induced by a low-protein diet, which may be the mechanism underlying reduced glomeruli [7]. Therefore, we investigated the role of apoptosis in the kidneys of rats with UPI-induced IUGR. Consistent with the aforementioned report of IUGR induced by protein restriction, our study revealed that the rats with IUGR had significantly higher cleaved caspase-3 (*p* < 0.05) and Bax/Bcl-2 levels (*p* < 0.001) than the control rats (see Additional file 1: Fig. S1 & Fig. 1).

### UPI induced metabolic profile changes in growth-restricted newborn rats

**Fig. 2:**
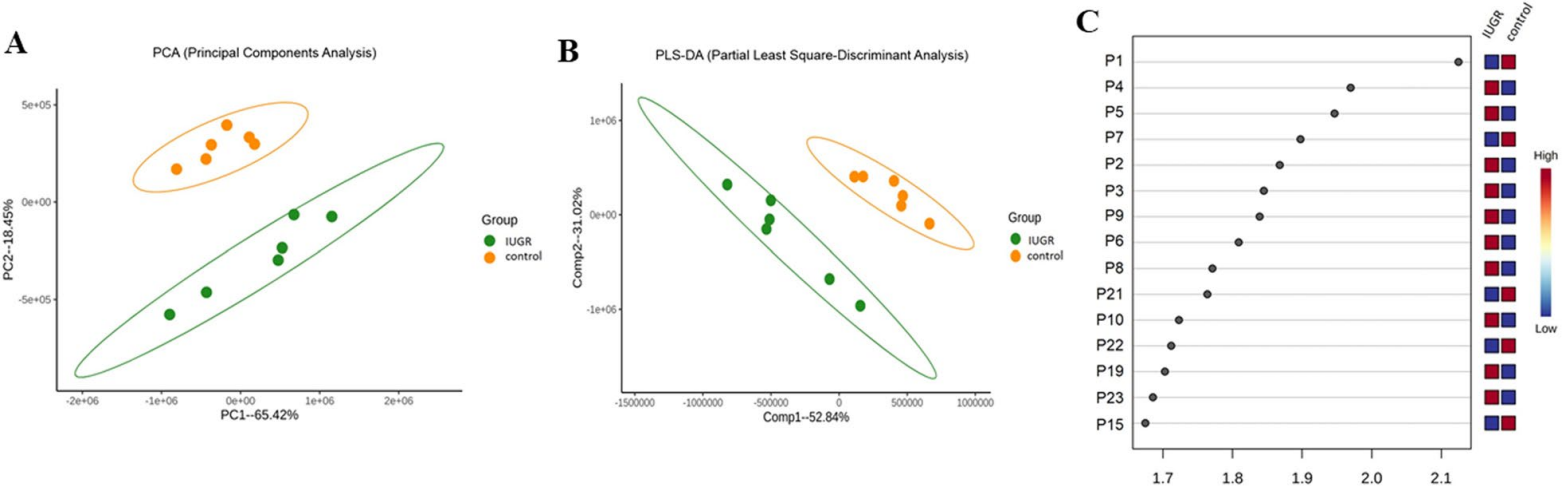
**A** Two-dimensional principal component analysis score plots. **B** Partial least-squares discriminant analysis (PLS-DA) score plots for principal components 1 and 2. The control group is represented by yellow circles, and the intrauterine growth restriction group is represented by green circles. **C** Compounds represented in the variable importance in projection scores are numbered according to their ranking in false discovery rate correction for univariate analysis. The 15 compounds that contributed most to group separation, as identified through PLS-DA and ranked by variable importance in projection scores. The heatmap on the right shows the mean intensity in the respective groups, with red and blue indicating high and low metabolite levels, respectively (n = 6)

Apoptosis leads to the penetration of the outer mitochondrial membrane and the release of enzymes involved in the regulation of metabolism in neonatal animals [9, 27]. Therefore, we investigated the metabolic and related pathways in the renal cortices of neonatal rats with IUGR through metabolomics. The PCA and PLS-DA score plots (Fig. 2A, B) obtained through multivariate statistical analysis of preprocessed spectral data revealed separation between the IUGR and control groups. The utilization of MS in negative ion mode demonstrated notable dissimilarities in the metabolite patterns between the IUGR group and the control group on postnatal day 7. The PCA scores of the renal cortical tissues of the control (orange in Fig. 2A) and experimental rats (green in Fig. 2A) were significantly different for the first two principal components. In the PCA, the first component accounted for 65.42% of the overall variance, and the second component accounted for 18.45% of the overall variability. The PLS-DA scores (Fig. 2B) exhibited statistically significant differences between the groups, suggesting notable alterations in the metabolic profiles of the pups affected by IUGR by the seventh day after birth. The PLS-DA model had an *R*^2^ value of 0.98 and *Q*^2^ value of 0.85. The PLS-DA VIP plot in Fig. 2C indicates that the differences between the IUGR and control groups were predominantly in taurine, acylcarnitine, glycerophospholipids, N-acetylglutamine, steroids, N-undecanoylglycine, ethyl glucuronide, and tryptophan.

**Fig. 3:**
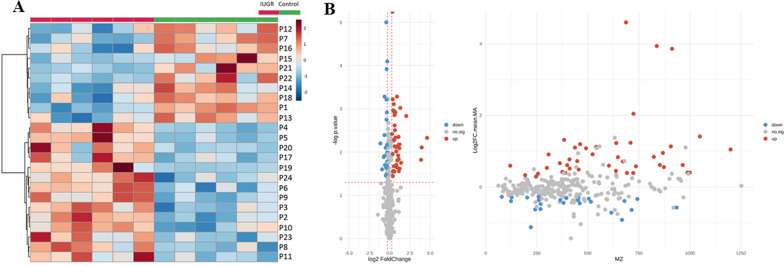
**A** Hierarchical clustering analysis identified 2 main clusters separating the control and IUGR kidney groups on postnatal day 7. The samples are clustered using Euclidean distance and Ward linkage. **B** Volcano plots of ultraperformance liquid chromatography–mass spectrometry (MS)/MS datasets. The *y*-axis indicates − log(*p*) values, whereas the *x*-axis indicates log_2_(fold change). Blue and red highlight significant metabolites (fold change > 1.2, *p* < 0.05). Gray dots indicate nonsignificant metabolites (n = 6)

We generated a heatmap through hierarchical clustering of metabolite concentrations by using standard Euclidean distance and Ward agglomeration. As shown in Fig. 3A, two large and distinct clusters formed. A volcano plot integrating FC and *p* values was employed to detect significant metabolites. Sixty-seven of the 237 analyzed metabolites differed significantly between groups (*p* < 0.05; Fig. 3B). On the basis of the normalized peak intensities, a two-sample *t* test with false discovery rate correction (*q* < 0.05; Table 2) revealed that 10 metabolites significantly differed between the IUGR and control groups. *Student’s t* test results corroborated the significantly elevated concentrations of acylcarnitine, oxidized phosphatidylglycerol, oxidized phosphatidylethanolamine, phosphatidylserine, steroids, and N-acetylglutamine in the IUGR group. In addition, the IUGR group had reduced concentrations of taurine, cysteine, phosphatidylethanolamine, and phosphatidylcholine compared with the control group.

**Table 2:** Ten compounds significantly different between the IUGR and control groups in the renal cortex of rats (n = 6) as determined through univariate analysis with false discovery rate correction (*q* < 0.05) on postnatal day 7

Compound	*p* value	*q* value	Identification	IUGR/control
P1	0.0001	0.0019	Taurine	Down
P2	0.0006	0.0238	PE(18:3(6Z,9Z,12Z)/P-18:1(9Z))	Down
P3	0.0011	0.0248	N-acetylglutamine	Up
P4	0.0011	0.0248	(2S)-2-hydroxy-2-(propan-2-yl)butanedioylcarnitine	Up
P5	0.0011	0.0248	PG(i-12:0/5-iso PGF2VI), PG(5-iso PGF2VI/i-12:0)	Up
P6	0.0012	0.0248	L-cysteine, D-cysteine	Down
P7	0.0012	0.0248	PC(15:0/18:2(9Z,12Z))	Down
P8	0.0014	0.0268	PS(DiMe(13,5)/MonoMe(13,5)), PS(MonoMe(13,5)/DiMe(13,5))	Up
P9	0.0015	0.0268	Cortisol, 18-hydroxycorticosterone, 17a,21-dihydroxy-5b-pregnane-3,11,20-trione	Up
P10	0.0024	0.0384	PE(22:0/PGJ2), PE(PGJ2/22:0)	Up

The ratio of IUGR to controls is either greater than 1 or less than 1, expressed as up or down. Compounds are numbered as P1–P10

### Metabolic pathway analysis

**Fig. 4:**
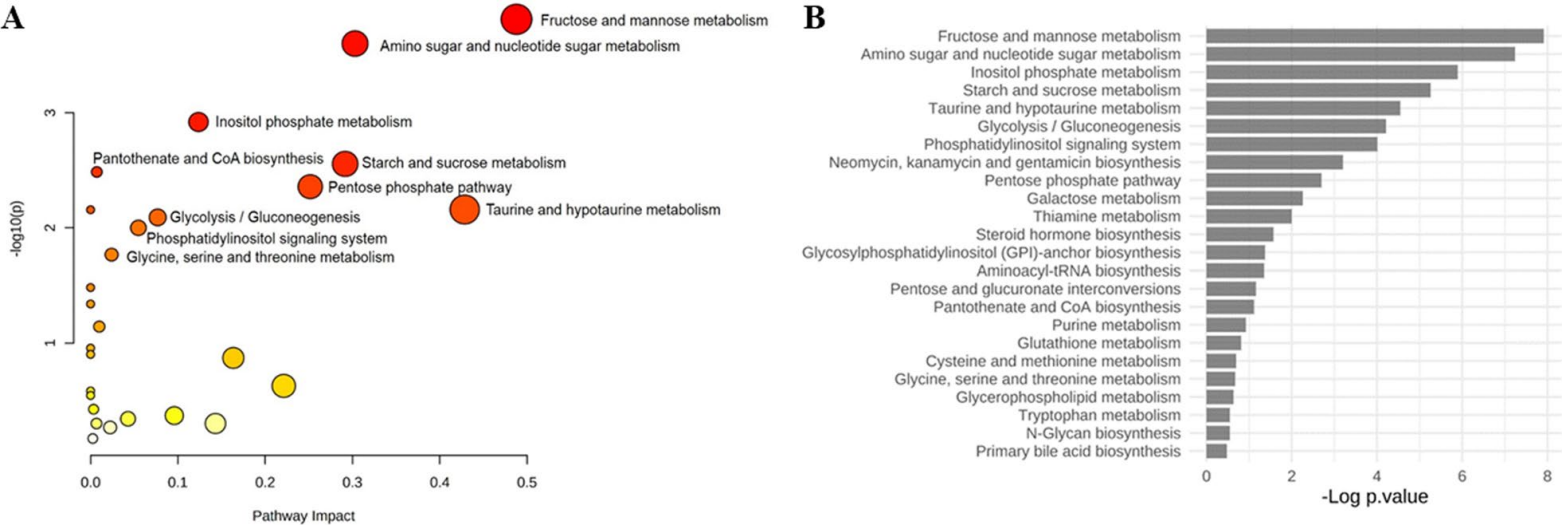
**A** Bubble plot and **B** bar chart of enrichment analysis results, emphasizing the metabolic pathways altered in the renal cortex of rats with IUGR on postnatal day 7 (n = 6)

To understand the pathways enriched for differential metabolites, KEGG pathway analysis was performed on the metabolites that were downregulated or upregulated > 1.2-fold in the IUGR group (compared with the control group). A total of 24 pathways were identified, of which three exhibited significant differences between the research groups (*q* < 0.05; Fig. 4 and Table 3). The primary modified metabolic pathways encompassed fructose and mannose metabolism, amino and nucleotide sugar metabolism, and inositol phosphate metabolism. In the IUGR group, the expression levels of mannose 6-phosphate, D-mannose 1-phosphate, and beta-D-fructose 6-phosphate were downregulated.

**Table 3:** Major differential metabolites and identified pathways according to the Kyoto encyclopedia of genes and genome on postnatal day 7 in the renal cortex of rats (*n* = 6)

Pathway	Match status	*p* value	*q* value	Impact	Metabolites
Fructose and mannose metabolism	4/18	0.0002	0.0105	0.4879	3a,6b,7a,12a-Tetrahydroxy-5b-cholanoic acid Mannose 6-phosphate D-mannose 1-phosphate Beta-D-fructose 6-phosphate
Amino and nucleotide sugar metabolism	5/37	0.0002	0.0105	0.3029	Mannose 6-phosphate D-mannose 1-phosphate Beta-D-fructose 6-phosphate Fructose 6-phosphate Glucose 1-phosphate
Inositol phosphate metabolism	4/30	0.0012	0.0337	0.1237	Glucose 6-phosphate D-myo-inositol 4-phosphate 1D-myo-inositol 3-phosphate Myo-inositol 1-phosphate

The compounds were identified using a process of comparing their *m/z* values and extracted ion chromatograms to the Human Metabolome Database. Additionally, manual verification was conducted to ensure matching *m/z* values and peak morphologies, as presented in Table 4.

**Table 4:** Signals detected by mass spectrometry were observed in the kidneys of rats on postnatal day 7 (*n* = 6)

Compound	Identification
P1	Taurine
P2	PE(18:3(6Z,9Z,12Z)/P-18:1(9Z))
P3	N-acetylglutamine
P4	(2S)-2-hydroxy-2-(propan-2-yl)butanedioylcarnitine
P5	PG(i-12:0/5-iso PGF2VI), PG(5-iso PGF2VI/i-12:0)
P6	L-cysteine, D-cysteine
P7	PC(15:0/18:2(9Z,12Z))
P8	PS(DiMe(13,5)/MonoMe(13,5)), PS(MonoMe(13,5)/DiMe(13,5))
P9	Cortisol, 18-hydroxycorticosterone, 17a,21-dihydroxy-5b-pregnane-3,11,20-trione
P10	PE(22:0/PGJ2), PE(PGJ2/22:0)
P11	dADP
P12	Inosine
P13	Dihydrothymine
P14	Galactose 1-phosphate, dolichyl phosphate D-mannose, fructose 1-phosphate, glucose 1-phosphate, D-mannose 1-phosphate, beta-D-fructose 2-phosphate, mannose 6-phosphate, glucose 6-phosphate, beta-D-glucose 6-phosphate, beta-D-fructose 6-phosphate, D-tagatose 6-phosphate, myo-Inositol 1-phosphate, D-myo-inositol 4-phosphate, myo-inositol 6-phosphate, D-myo-inositol 3-phosphate, fructose 6-phosphate, sorbose 1-phosphate
P15	L-tryptophan, D-tryptophan, ( ±)-tryptophan
P16	PE(16:1(9Z)/18:1(11Z)), PE(16:1(9Z)/18:1(9Z)), PE(18:1(9Z)/16:1(9Z)), PE-NMe2(14:1(9Z)/18:1(9Z)), PE-NMe2(14:1(9Z)/18:1(11Z)), PE-NMe2(18:1(11Z)/14:1(9Z)), PE-NMe2(18:1(9Z)/14:1(9Z))
P17	PI(20:1(11Z)/20:4(8Z,11Z,14Z,17Z)-2OH(5S,6R)), PI(20:4(8Z,11Z,14Z,17Z)-2OH(5S,6R)/20:1(11Z)), PI(22:4(10Z,13Z,16Z,19Z)/18:1(12Z)-2OH(9,10)), PI(18:1(12Z)-2OH(9,10)/22:4(10Z,13Z,16Z,19Z))
P18	Pancrelipase
P19	PA(22:4(7Z,10Z,13Z,16Z)/6keto-PGF1alpha), PA(22:4(7Z,10Z,13Z,16Z)/TXB2), PA(6keto-PGF1alpha/22:4(7Z,10Z,13Z,16Z)), PA(TXB2/22:4(7Z,10Z,13Z,16Z))
P20	7-Hydroxy-3-oxocholanoic acid, 7alpha-hydroxy-3-oxo-5beta-cholan-24-oic acid, 12alpha-hydroxy-3-oxo-5beta-cholan-24-oic acid
P21	N-undecanoylglycine
P22	Ethyl glucuronide
P23	PC(18:1(11Z)/TXB2), PC(TXB2/18:1(11Z)), PC(18:1(9Z)/TXB2), PC(TXB2/18:1(9Z))
P24	CarnocinCP5

Compounds are numbered as P1–P24

## Discussion

This study is the first to explore the differences in the renal metabolome and altered metabolic pathways in rats with IUGR. We successfully established an animal model of UPI-induced IUGR through bilateral uterine vessel ligation. The IUGR group had a substantially lower average birth weight than the control group. In addition, the metabolite profiles and metabolic pathways of the renal cortices of the rats with IUGR were significantly different from those of the control rats, which could be grouped using metabolomics and high-throughput processing. The IUGR group had substantially higher levels of acylcarnitine, oxidized phosphatidylglycerols, oxidized phosphatidylethanolamine, phosphatidylserines, steroids, and N-acetylglutamine than the control group. In contrast, in the IUGR group, taurine, cysteine, phosphatidylethanolamine, and phosphatidylcholine were significantly reduced. The most discriminative metabolic pathways in the renal cortex of rats with IUGR were those related to fructose and mannose, amino and nucleotide sugars, and inositol phosphate.

This study revealed that UPI-induced IUGR rats had substantially lower kidney weight on postnatal day 7 compared to control rats. However, this difference was not observed after adjusting for body weight; this result is consistent with previous findings [28, 29]. Weight for gestational age has a significant positive effect on kidney size [30, 31], possibly because IUGR causes a low nephron number [32]. But IUGR not only affects kidney size, it can also reduce overall growth and cause weight loss. We believe this may explain why there was no significant difference in kidney weight between groups after adjusting for body weight. Further studies are needed to clarify the relationship between IUGR and kidney size at different stages.

In this study, significantly increased cleaved caspase-3 and Bax/Bcl-2 levels indicated increased cell apoptosis in newborn rats with UPI-induced IUGR that affected at least their intrinsic mitochondrial apoptosis pathways in the renal cortex. Collaborative activation of the proapoptotic Bcl-2 and caspase families during apoptosis results in the penetration of the outer mitochondrial membrane and release of enzymes involved in regulating metabolism [9].

In this study, rats with IUGR exhibited elevated concentrations of acylcarnitine, oxidized phosphatidylglycerol, oxidized phosphatidylethanolamine, phosphatidylserine, steroids, and N-acetylglutamine. Acylcarnitines are derived from mitochondrial acyl-coenzyme A (CoA) metabolism, and the accumulation of acylcarnitines can interfere with insulin signaling in type II diabetes [33, 34] and thus may be associated with higher risks of impaired glucose tolerance and type II diabetes in IUGR. Acylcarnitines are also crucial for energy metabolism because they are involved in fatty acid oxidation [35]. The accumulation of acylcarnitines and fatty acids has been reported in umbilical cord blood [15] and the blood of neonates [36] in IUGR. In comparison to the control group, the IUGR group exhibited significant increases in metabolites such as phosphatidylserine and N-acetylglutamine on both postnatal day 0 and postnatal day 7 (postnatal day 0 data not provided). Conversely, phosphatidylethanolamine showed a significant decrease in the IUGR group. These metabolites could potentially serve as early indicators of IUGR resulting from UPI. The decline in levels of taurine and cysteine, together with the elevation of acylcarnitines, has been observed to disrupt fatty acid metabolism and lead to an increase in oxidized glycerophospholipid during the end stages of nephrogenesis in IUGR.

Another metabolite, N-acetylglutamine, is a mitochondrial intermediate whose concentration increases in response to protein consumption. N-acetylglutamine is a uremic toxin in high serum or plasma concentrations [37, 38]. N-acetylglutamine can be released from peptides by an N-acylpeptide hydrolase or can be biosynthesized from L-glutamine and acetyl-CoA by glutamine N-acyltransferase [39]. Increased urinary N-acetylglutamine in patients treated with aminoglycosides or glycopeptides indicates renal tubular injury.

In this study, rats with IUGR had substantially lower concentrations of taurine, cysteine, phosphatidylethanolamine, and phosphatidylcholine in their renal cortices than control rats. Taurine has proliferative, cytoprotective, and anti-inflammatory properties and is involved in the modulation of intracellular calcium levels, osmoregulation, and neurodevelopment; it is the most abundant free amino acid produced from methionine, serine, and their precursor cysteine [40]. Taurine also binds bile acids, thereby improving their detergency and solubility and reducing reabsorption [41]. In addition, previous studies have demonstrated significantly lower levels of taurine in placentas as well as the urine and serum samples of neonates with IUGR [42–44].

Cysteine is necessary for the synthesis of CoA, which is essential for cellular oxidative pathways such as the Krebs cycle, amino acid oxidation, fatty acid oxidation, protein modification, and lipid synthesis [45]. The reductions in taurine and cysteine in IUGR may interfere with the oxidation of amino and fatty acids, thereby impairing the TCA cycle and protein and lipid synthesis.

In this study, we found an altered lipid profile in IUGR, including higher levels of oxidized phosphatidylglycerol, oxidized phosphatidylethanolamine, and phosphatidylserine and lower levels of phosphatidylethanolamine and phosphatidylcholine. As we found in the renal cortex of IUGR rats, oxidized phosphatidylethanolamine is significantly more abundant in the blood of mothers with severe preeclampsia [46]. Phosphatidylserine and phosphatidylcholine are procoagulant phospholipids derived from activated platelets and cause placental insufficiency associated with a phospholipid-induced hypercoagulable state in the placental circulation [47]. Phosphatidylcholine is also a major component of cell membranes and a pulmonary surfactant and plays a key role in membrane-mediated cell signaling. However, the associations between phosphatidylcholine levels in cord blood samples and IUGR have been inconsistent [48, 49]. Our findings indicate that IUGR causes the abnormal catabolism of proteins and lipids, and we speculate that substrate accumulation contributes to subsequent metabolic disease. Additional studies are required to delineate the role of such substrates in renal metabolism.

We identified major pathways altered in IUGR, including those responsible for the metabolism of fructose and mannose, amino and nucleotide sugars, and inositol phosphate. Fructose and mannose metabolism plays a key role in fetal growth and development [50]. Enzymatic function in fructose metabolism may indicate metabolic prognosis. Untreated abnormal fructose metabolism, including hypoglycemia, metabolic acidosis, and glycosylation deficiency, contributes to kidney injury [51].

IUGR activates a series of adaptive mechanisms to increase the chances of survival and saves glucose to ensure the nutrition of vital organs, thereby reducing insulin secretion. Decreased fetal insulin production results in inositol excretion from the intracellular compartment to the extracellular compartment, causing intracellular depletion [51, 52]. Other studies have demonstrated that members of the inositol phosphate metabolism pathway, which was significantly affected in the renal cortex of the IUGR pups in our study, regulate insulin signaling, phosphoinositide 3-kinase/protein kinase B signaling, endocytosis, vesicle trafficking, and cell migration, proliferation, and apoptosis [53].

Using an IUGR animal model, we previously discovered that the metabolism of amino and nucleotide sugars, which is essential for energy production, was downregulated [54]. We discovered that all perturbed metabolic pathways affected the TCA cycle. Furthermore, we found increased apoptosis through the intrinsic pathway in cells of the renal cortex in IUGR. Increased apoptosis in the renal cortex in IUGR may be caused by the disruption of the TCA cycle due to the glycolytic metabolic pathway.

There are several limitations to this study. We used only the kidney tissue from rats with IUGR and did not analyze the urine or blood samples to investigate physiological dynamics. However, we aimed to exclude possible errors and to determine the specific effect of UPI on the renal metabolomic profile of offspring by performing bilateral uterine vessel ligation on mothers. Furthermore, this study's sample size is relatively limited. This research was informed by prior studies conducted on IUGR rats [55]. With α and β levels established at 0.05 and 0.20, respectively, it was ascertained that the sample size was adequate for identifying significant effects. Third, the results of this study must be extrapolated to humans with caution, as they were obtained through an animal experiment.

In conclusion, UPI alters renal metabolism and induces renal cell apoptosis in neonatal rats with growth restriction. Our findings have the potential to increase our knowledge of novel biomarkers and metabolic pathways implicated in IUGR-related renal changes, providing an explanation for increased susceptibility to renal disease in adulthood.

### Supplementary Information

**Additional file 1**. **Figure S1**. All western blot bands for cleaved caspase-3, Bax, Bcl-2, and the loading control in the control and uteroplacental insufficiency induced intrauterine growth restriction rats

## Data Availability

The datasets used and/or analysed during the current study are available from the corresponding author on reasonable request.

## Abbreviations

CoA: Coenzyme A
FC: Fold change
IUGR: Intrauterine growth restriction
KEGG: Kyoto Encyclopedia of Genes and Genomes
MS: Mass spectrometry
PBS: Phosphate-buffered saline
PCA: Principal component analysis
PLS-DA: Partial least-squares discriminant analysis
UPI: Uteroplacental insufficiency
UPLC: Ultraperformance liquid chromatography
VIP: Variable importance in projection
