# Supplementary material for: Intrauterine growth restriction alters kidney metabolism at the end of nephrogenesis
Source: Nutr Metab (Lond). 2023 Nov 21;20:50. doi: 10.1186/s12986-023-00769-6 (PMC10664663; doi:10.1186/s12986-023-00769-6)

Figure S1

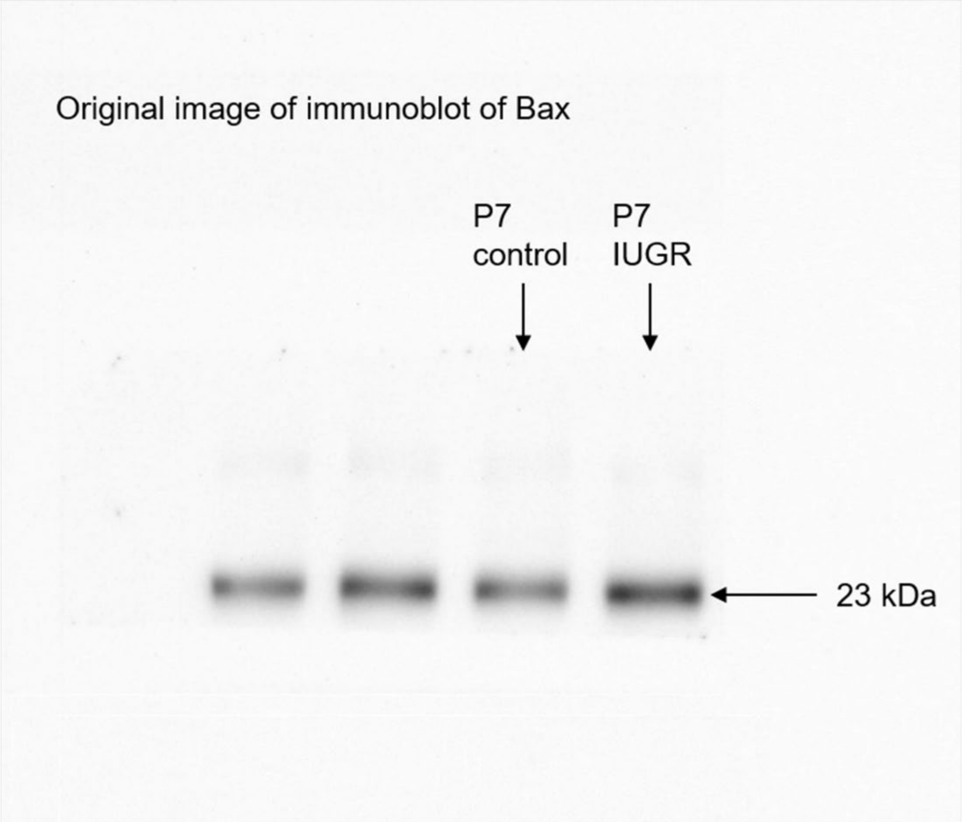
Original image of immunoblot of Bax
P7
control
P7
IUGR
23 kDa

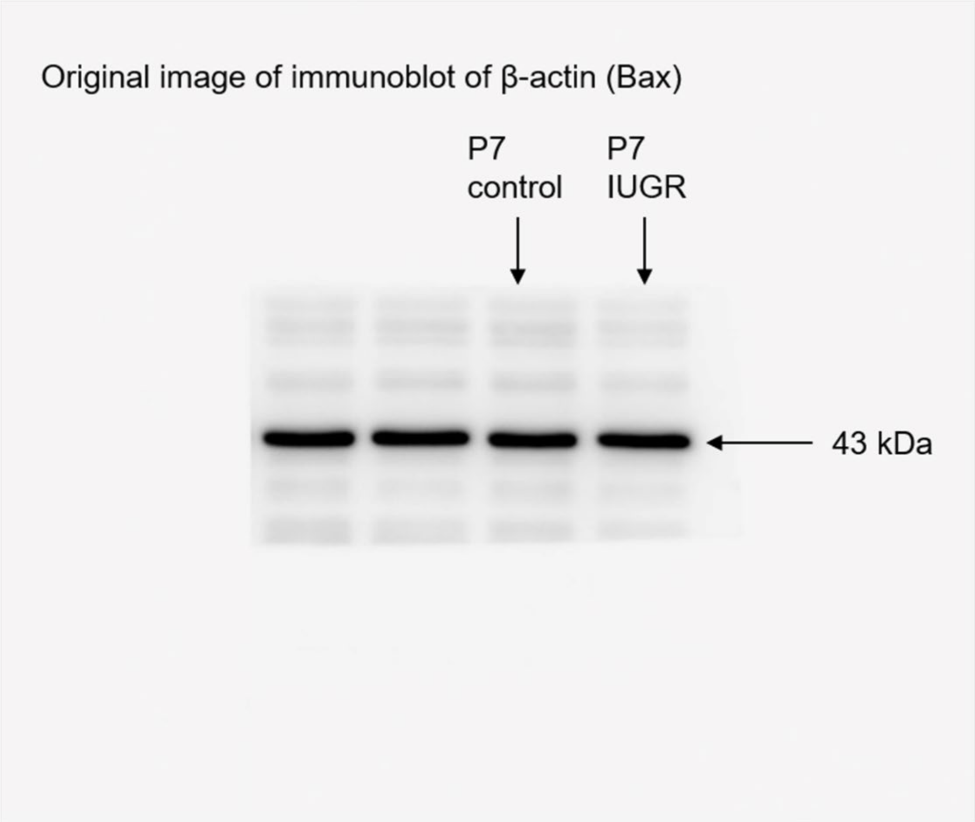
Original image of immunoblot of β-actin (Bax)
P7
control
P7
IUGR
43 kDa

Original image of immunoblot of Bax

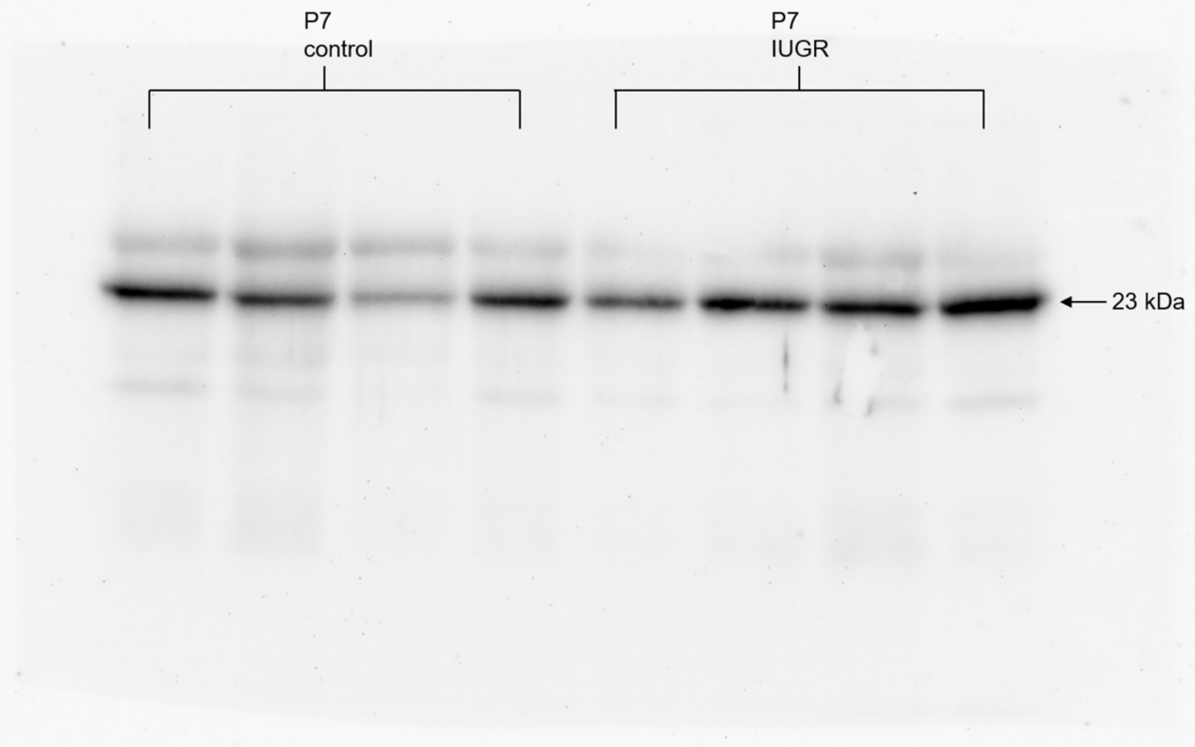

Original image of immunoblot of β-actin (Bax)

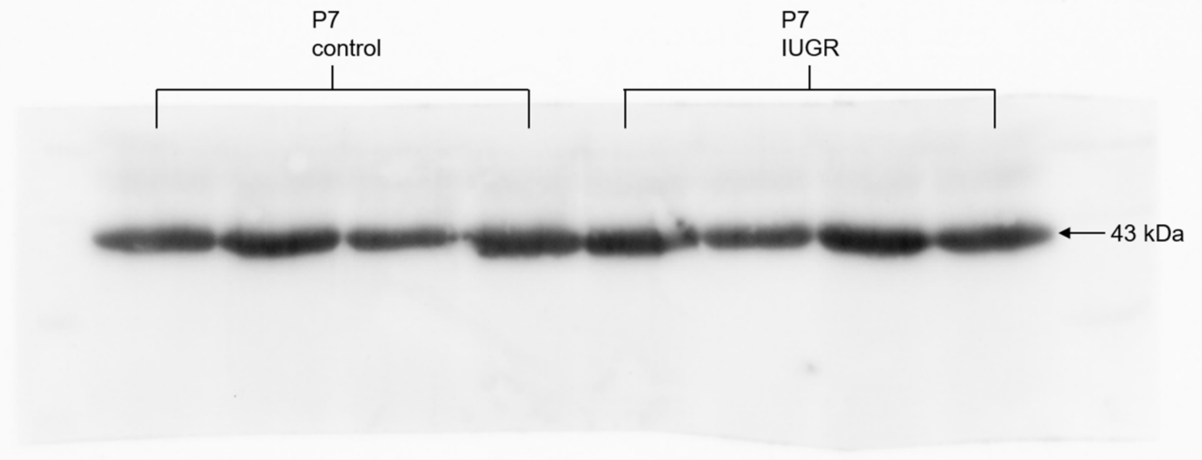

Original image of immunoblot of Bcl-2

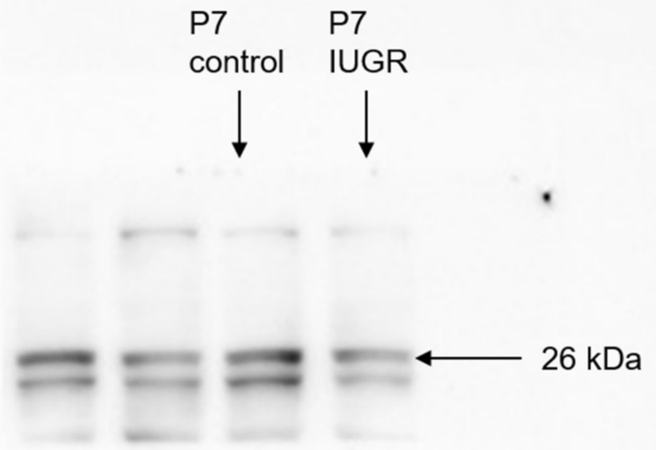

Original image of immunoblot of β-actin (Bcl-2)

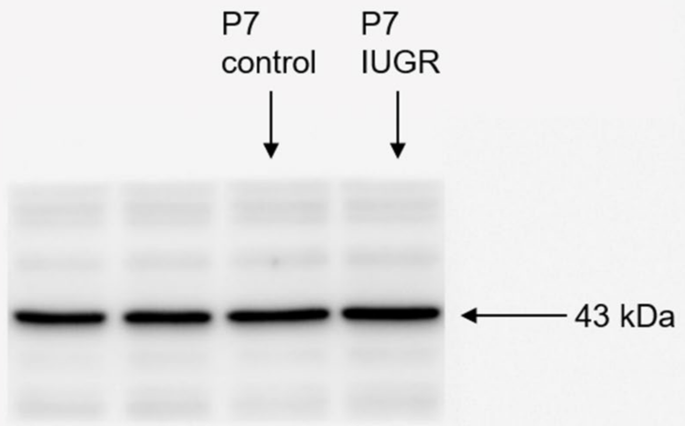

Original image of immunoblot of Bcl-2

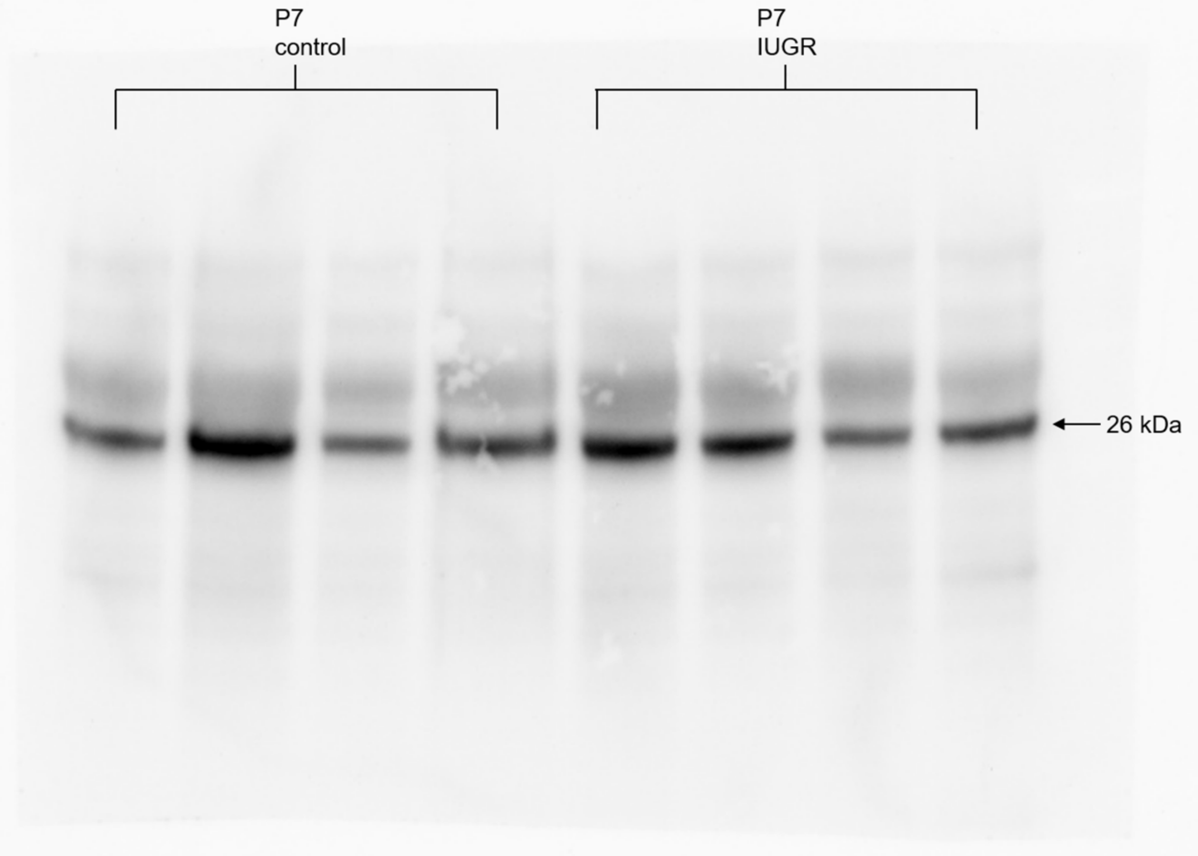

Original image of immunoblot of β-actin (Bcl-2)

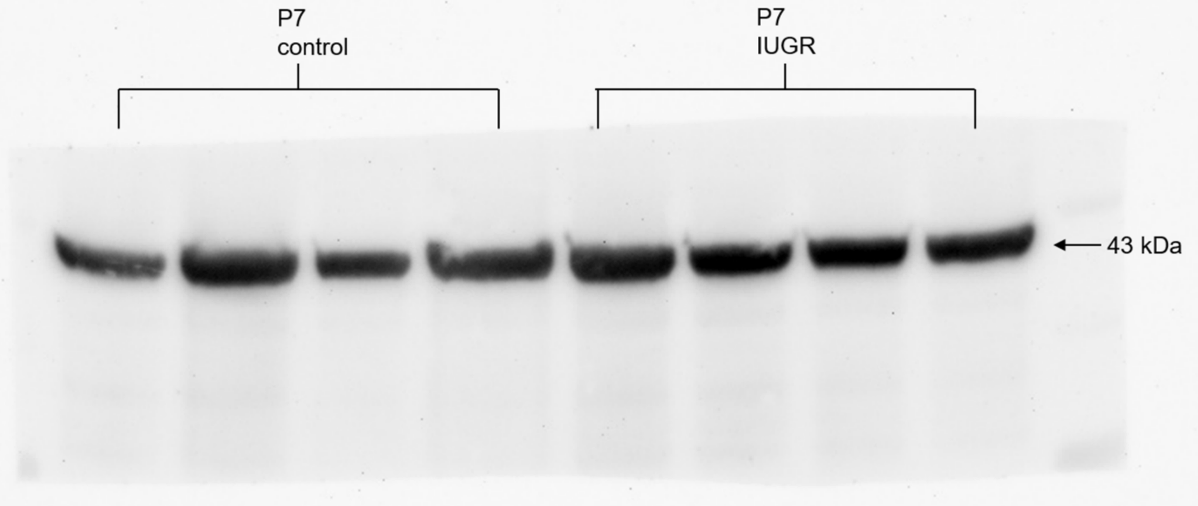

Original image of immunoblot of cleaved caspase-3

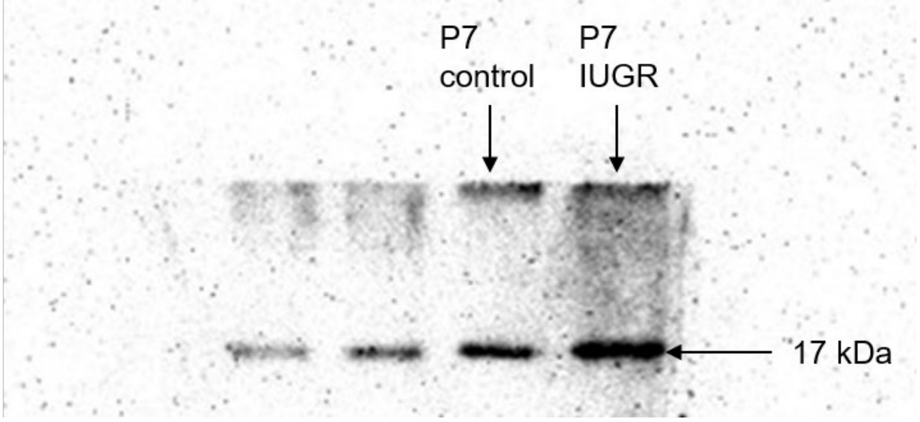

Original image of immunoblot of β-actin (cleaved caspase-3)

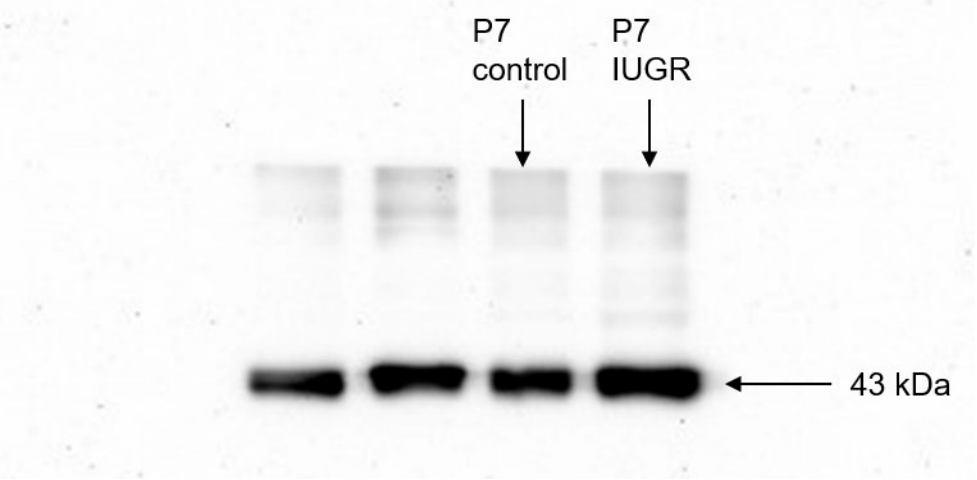

Original image of immunoblot of cleaved caspase-3

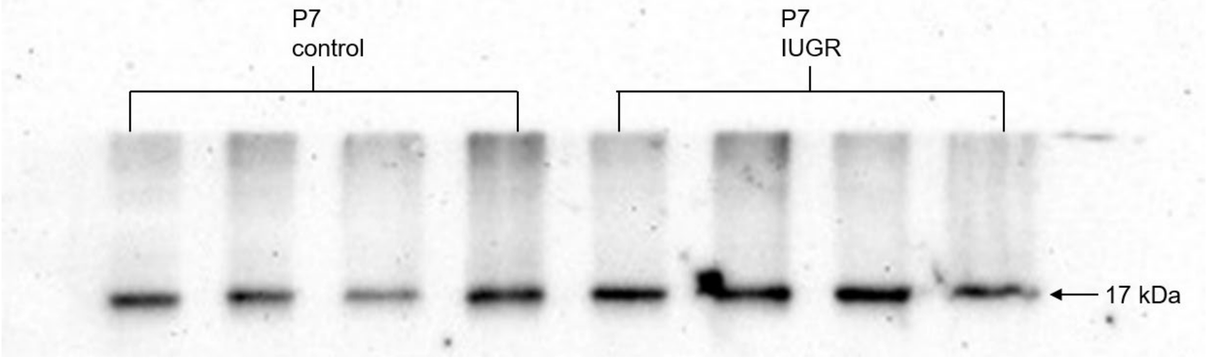

Original image of immunoblot of β-actin (cleaved caspase-3)

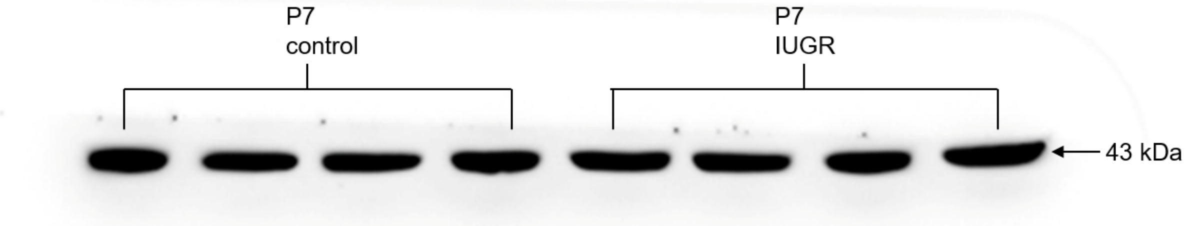

Original image of immunoblot of cleaved caspase-3

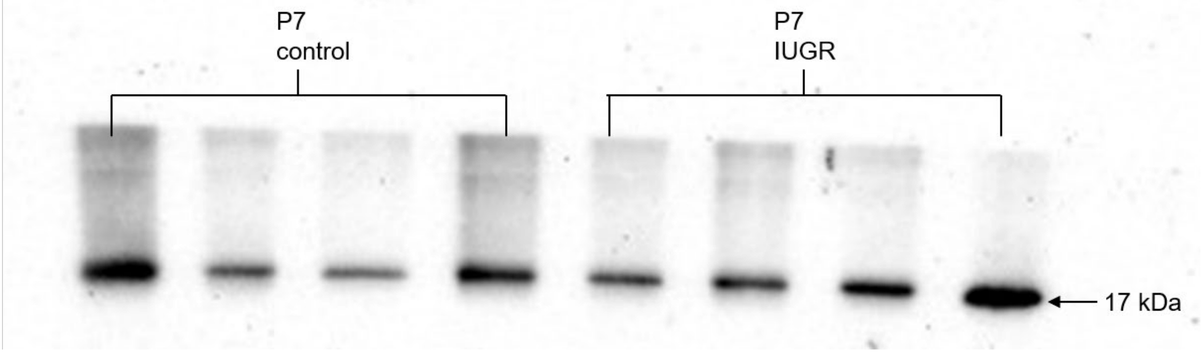

Original image of immunoblot of β-actin (cleaved caspase-3)

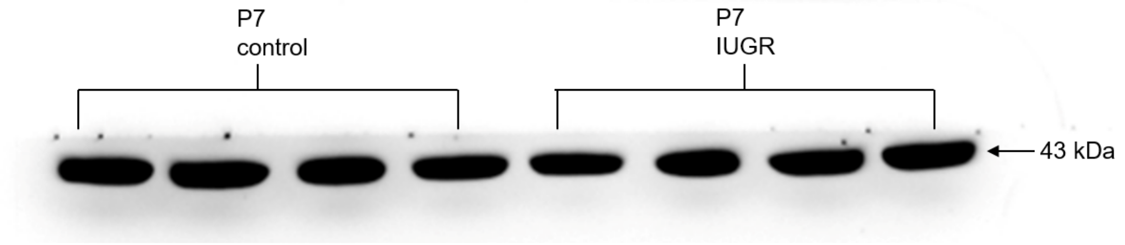

Supplement: Supplementary file 1 — Additional file 1. Figure S1. All western blot bands for cleaved caspase-3, Bax, Bcl-2, and the loading control in the control and uteroplacental insufficiency induced intrauterine growth restriction rats [file 12986_2023_769_MOESM1_ESM.pdf]
